# *Aedes aegypti* Mosquitoes Imported into the Netherlands, 2010

**DOI:** 10.3201/eid1712.110992

**Published:** 2011-12

**Authors:** Julia E. Brown, Ernst-Jan Scholte, Marian Dik, Wietse Den Hartog, Jacob Beeuwkes, Jeffrey R. Powell

**Affiliations:** Yale University, New Haven, Connecticut, USA (J.E. Brown, J.R. Powell);; Dutch National Center for Monitoring of Vectors, Wageningen, the Netherlands (E.J. Scholte, M. Dik, W. Den Hartog, J. Beeuwkes)

**Keywords:** vector-borne infections, Aedes aegypti, mosquitoes, dengue, yellow fever, virus, invasion, used tires, the Netherlands, imported, global health

## Abstract

During summer 2010, *Aedes aegypti* mosquitoes were discovered in the Netherlands. Using genetic markers, we tracked the origin of these mosquitoes to a tire shipment from Miami, Florida, USA. Surveillance of tire exports from the United States should be included as part of a comprehensive surveillance system.

During summer 2010, national surveillance activities detected *Aedes aegypti* mosquitoes in 2 tire yards in the Netherlands (*1*,*2*). *Ae. aegypti* mosquitoes are the principal worldwide vectors of dengue and yellow fever viruses, which cause a wide range of illnesses varying from asymptomatic to life threatening (*3*). Typically, these mosquitoes are found in tropical and subtropical regions throughout the world and had not been found in Europe since they were eliminated in the region shortly after World War II (*3*).

In the Netherlands, a tire shipment from southern Florida, USA, was identified as a potential source of *Ae. aegypti* mosquitoes (*1*,*2*). Tires were received from Miami, Florida, USA, at the 2 affected tire yards during the months before the discovery. Tire transportation has not been considered to play a large role in recent invasions of *Ae. aegypti* mosquitoes, as it has been for the Asian tiger mosquito, *Ae. albopictus* (*4*). However, several decades ago, tires from the United States were implicated as a source of *Ae. aegypti* mosquitoes transported to Central and South America after abandonment of the *Ae. aegypti* mosquito eradication program (*5*).

Effective vector control and prevention measures require knowledge of the origin of invasive mosquitoes and how they are transported. Therefore, we set out to determine the origin of the *Ae. aegypti* mosquitoes in the Netherlands by using a genetic approach.

## The Study

Previous work in our laboratory validated a set of 12 microsatellite markers to distinguish between global populations of *Ae. aegypti* mosquitoes (*6*). We screened these markers in 8 mosquito specimens from the 2010 invasion in the Netherlands and compared their genotypes with those from 736 *Ae. aegypti* mosquito specimens from 15 reference populations around the world, including 4 Florida locations.

We analyzed 8 mosquitoes from 2 tire yards in the Netherlands, 2 mosquitoes from site 1 and 6 from site 2 (*2*). The samples consisted of individual legs preserved in 70% ethanol. These samples were compared with previously screened *Ae. aegypti* mosquito populations from 14 locations worldwide: Palm Beach County, Vaca Key, and Conch Key, Florida, USA; Houston, Texas, USA; Pijijiapan and Coatzacoalcos, Mexico; Dominica; Bolivar and Zulia, Venezuela; Rayong and Prachuabkhirikan, Thailand; Tahiti, French Polynesia; and Cairns and Townsville, Queensland, Australia. The number of mosquitoes analyzed per reference population is indicated in Brown et al. (*6*). We also included in the analyses 47 newly acquired *Ae. aegypti* mosquito samples from Miami. Collection methods are described elsewhere (*2*,*6*).

Genomic DNA was extracted from each mosquito by using DNeasy kits (QIAGEN, Valencia, CA, USA). The samples from the Netherlands and Miami were screened for variation at 12 microsatellite loci following published methods (*6*,*7*). Chord distances between each pair of populations were calculated in GENETIX (*8*) and used in 2 distance-based cluster analyses: a principal components analysis using PAST (*9*) and a neighbor-joining network using MEGA4 (*10*). The Bayesian clustering algorithm in the program STRUCTURE (*11*) was used to identify genetic clusters and assign individual mosquitoes to these clusters with no a priori information about sampling locations. To determine the best genetic match for samples from the Netherlands, we conducted 5 independent runs for each assumed number of populations, K, 1–17. For all runs, we assumed an admixture model and correlated allele frequencies and used a burn-in value of 100,000 iterations followed by 500,000 replications. Results from STRUCTURE were visualized using DISTRUCT (*12*). A group assignment test was implemented in GENECLASS2 (*13*) to assign the mosquitoes in the Netherlands of unknown origin back to the reference populations with relative probabilities.

Population-level (Figure, panels A, B) and individual-level (Figure, panel C) analyses suggest that the *Ae. aegypti* mosquito samples from the Netherlands are in the same genetic group as populations from southern Florida. Among these Florida populations, the group assignment test (*13*) identified Miami as the likely source of the samples from the Netherlands, with a relative probability of 100% compared with the other 14 reference populations. The recorded import of tires from the Miami area to the sites in the Netherlands where *Ae. aegypti* mosquitoes were discovered strongly corroborates the results from our genetic data, clearly indicating introduction of *Ae. aegypti* mosquitoes from Miami.

**Figure:**
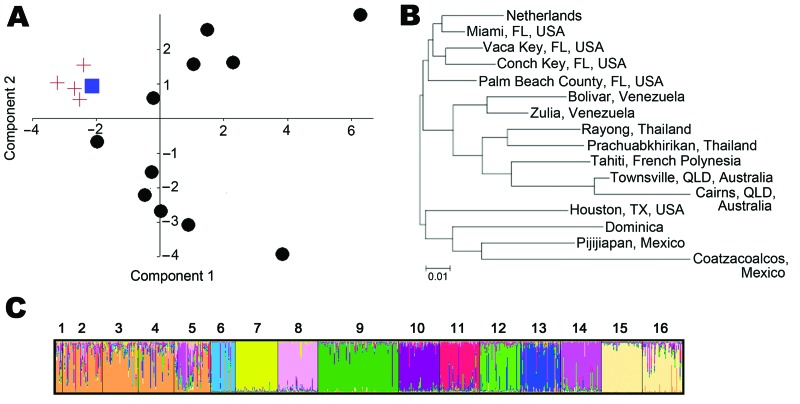
A) Principal components analysis based on pairwise population chord distances. The *Adedes aegypti* mosquito population in the Netherlands is represented by a blue square, the Florida, USA, populations by red crosses, and all other populations by black circles. B) Neighbor-joining network based on chord distances. QLD, Queensland. Scale bar indicates nucleotide substitutions per site. C) Individual mosquito–based Bayesian cluster analysis (K = 11) of the *Ae. aegypti* mosquito samples from the Netherlands and 15 reference populations. Populations are labeled as follows: 1, the Netherlands; 2, Miami, Florida, USA; 3, Vaca Key, Florida, USA; 4, Conch Key, Florida, USA; 5, Palm Beach County, Florida, USA; 6, Houston, Texas, USA; 7, Coatzacoalcos, Mexico; 8, Pijijiapan, Mexico; 9, Dominica; 10, Bolivar, Venezuela; 11, Zulia, Venezuela; 12, Rayong, Thailand; 13, Prachuabkhirikan, Thailand; 14, Tahiti, French Polynesia; 15, Cairns, Queensland, Australia; 16, Townsville, Queensland, Australia.

## Conclusions

Our findings suggest that 1 of the world’s most dangerous vector arthropods entered Europe through a tire shipment from Miami. Although the importation of mosquitoes into the United States through the used tire trade has received considerable focus, our results indicate that equal caution should be exercised when tires are exported out of the southern United States, particularly into regions where *Ae. aegypti* mosquitoes are absent. Because vector exportation from the United States has now occurred multiple times (*5*,*14*), tires should be included as part of a comprehensive surveillance system to prevent future incidents.

Given the recent reemergence of dengue fever in Florida (*15*), we know that populations of *Ae. aegypti* mosquitoes from that region are fully capable of causing outbreaks of arboviral diseases. In the temperate climate of northern Europe, the epidemiologic risk is higher during the warm summer months, when viruses could be introduced to these new vector populations by travelers from tropical locations. This scenario would likely require close human–mosquito interactions at the site of the introductions. Overall, the risk is much greater in southern Europe, where the climate allows for year-round establishment of *Ae. aegypti* mosquito populations (*3*). Vector surveillance will prove crucial to prevent reinvasion of the region by this species of mosquitoes. In addition, cooperation between government scientists, policy makers, and companies involved in international trade is necessary domestically and internationally to determine the origins of exotic mosquito vector invasions, rather than fighting diseases as they occur.
